# Individual socioeconomic and neighborhood factors predict changes in sports activity during the transition to retirement

**DOI:** 10.1186/s11556-021-00268-8

**Published:** 2021-07-03

**Authors:** Dorothee Jürgens, Benjamin Schüz

**Affiliations:** University of Bremen, Institute of Public Health and Nursing Research, Grazer Str. 4, 28359 Bremen, Germany

**Keywords:** Sports activity, Physical activity, Social inequality, Retirement, Social-ecological model, Older adults

## Abstract

**Background:**

There are substantial socioeconomic status (SES) differences in sports activity (SA) during the transition to retirement. In line with social-ecological models, the aim of this longitudinal study was to examine the association of perceptions of social and physical neighborhood factors with changes in SA across the retirement transition and to examine potential interactions with SES factors.

**Methods:**

Data from 6 waves of the German Ageing Survey (DEAS) provided 710 participants (at baseline: mean age 61.1, 52.9% of men) who retired between baseline (1996, 2002, 2008, 2011) and their 6-year follow-up assessment. Associations between changes in SA (increases and decreases compared to retaining) and individual SES and neighborhood factors were estimated using multinomial logistic regression analysis.

**Results:**

Increases were observed in 18.45% of participants, decreases in 10%. Occupational prestige was a risk factor for decreases, education a resource for increases in SA. Interactions between household income and several neighborhood factors were observed.

**Conclusions:**

In line with social-ecological models, individual, neighborhood factors and interacting associations were found. In particular safety perceptions could be a resource for promotion SA in older adults who experience disadvantage.

## Background

Similar to many countries in the Global North, Germany is experiencing an increase in the proportion of older adults and an increase in general life expectancy [1]. Over the next decades, the baby boomer generation will change the population structure as this group will reach retirement age [2]. This poses major challenges for social security systems, implicating the necessity of research into modifiable determinants of health such as physical activity (PA) [3]. The transition period into retirement is an important time of change [4], and it is associated with significant life changes [5] – also affecting changes in behaviors such as PA and sports activity (SA) [6, 7]. Retirement age is mandatory in Germany, thus the transition to retirement is a standardized marking point that can be foreseen and planned [5]. These aspects can be used for health promotion and prevention to plan and implement interventions.

SA is a key component of active and healthy aging [8] and constitutes an important protective factor against the development of several chronic diseases associated with aging such as type 2 diabetes, cardiovascular diseases, weight gain, cognitive impairments and depression, and is associated with lower all-cause mortality [9–12]. Furthermore, engaging in SA has the potential to facilitate social interactions that promote autonomy, self-confidence and quality of life [13, 14], and the change in daily routines during the transition to retirment provides more opportunities for SA [15, 16]. However, studies on the transition period to retirement note that the loss of work-related and transport-related activity is not adequately compensated by leisure-time PA, resulting in declines in overall activity [15, 17, 18]. Consequently, research on the potentially modifiable determinant of SA changes during the transition to retirement is of key importance.

These potentially modifiable determinants of SA can be organized within social ecological models [19]. Such models describe the determinants of health and health behaviors on multiple levels, integrating (i) individual determinants such as socioeconomic status (SES), health status, age, or psychosocial factors, (ii) factors relating to the social environment such as social support and social cohesion (iii) broader environmental factors including the built environment (e.g., PA facilities, security perceptions), and (iv) societal factors such as the structure of the health care system [19]. Applying social -ecological models in public health research allows concurrently examining individual- and context-level determinants as well as interactions between determinants on different levels [19–21]. For example, a study on a previous wave of the current data set [22] found that the effects of individual-level determinants of PA such as behavioral plans were modified by financial resources on the level of administrative districts: There were stronger effects in individuals living in districts with more resources, even after controlling for individual-level financial resources. Some previous studies point to the importance of perceptions of the physical and social environment as determinants for activity upon retirement with more favorable perceptions of facilities and safety [23–25] and closer social affiliation [26] related to higher levels of activity. A systematic review [27] emphasized a significant association of sports facilities on activity in older adults.

However, apart from a smaller longitudinal study in Belgium [28], relatively little is known about the association of perceptions of neighborhood factors on leisure-time PA implied in social ecological models during the retirement transition.

This study therefore has two main aims: First, to examine the role of perceptions of the physical and social neighborhood indicators on increases and decreases in SA during the retirement transition. Second, as previous studies have shown that changes in SA upon retirement differ according to individual SES indicators [17, 29, 30], a further aim was to identify whether the association of environmental indicators on changes in SA varies between socio-economic groups. Here, we examined potential interactions between individual SES indicators based on the PROGRESS-Plus framework [31] and environmental indicators.

## Methods

This study uses data from the German Aging Survey (DEAS), an ongoing population-based long-term study of community-dwelling adults over 40 living in Germany [2]. In a cohort-sequential design, since 1996, a large-scale baseline sample is drawn every 6 years and followed up over time [32]. The survey uses random sampling stratified by sex, age group, and region of residence (former East vs West Germany) based on the population register. The survey combines a personal oral interview and a self-reported questionnaire and covers a broad range of aspects of living conditions and a variety of age-related topics [33].

**Fig. 1:**
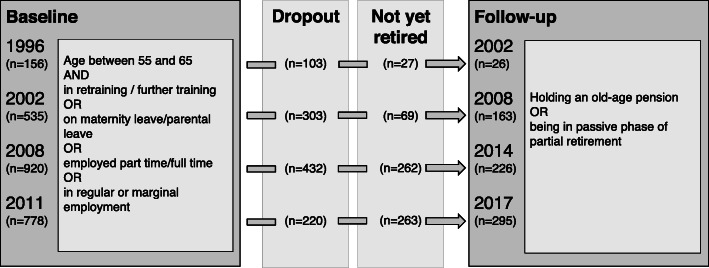
Sampling strategy

For the present analysis, we considered 710 participants who transitioned into retirement between their baseline assessments in 1996/2002/2008/2011 and the follow-up assessment after 6 years 2002/2008/2014/2017. Figure 1 outlines the sampling strategy. Inclusion criteria were being aged between 55 and 65 years and being employed or in retraining or in parental leave at baseline, and indicating retirement at the respective follow-up assessment.

### Variables and measurement

#### Sports activity

SA was assessed using a single item ‘How often do you do sports, such as hiking, soccer, gymnastics, or swimming?’, and responses ranged from daily [1] to never [6] [33]. In line with previous studies [34, 35], a dichotomized variable was created to differentiate between individuals who were active (response options ‘daily’, ‘several times a week’ and ‘once a week’) or inactive (response options ‘between 1 to 3 times a month’, ‘less often’ and ‘never’). Based on the SA levels at the follow-up assessment, four patterns of SA change were identified: (i) People who were physically inactive at baseline and follow-up assessment (remained inactive), (ii) persons who changed their SA from active at baseline to inactive at follow-up assessment (became inactive), (iii) persons who were active at baseline and follow-up assessment (remained active), (iv) persons who changed their SA from inactive at baseline to active at follow-up assessment (become active).

#### Indicators of social and physical environment

Environmental perceptions (safety, shopping facilities, health facilities, positive memories and feelings of attachment) were assessed with single items such as. “There are enough doctors and pharmacies in the neighborhood” (health facilities) or “I feel attached to the neighborhood” (feelings of attachment). Environmental perceptions were assessed on a four-point scale (1 = strongly agree, 2 = agree, 3 = disagree, 4 = strongly disagree) in wave 3 (year 2008) and wave 4 (years 2011), but dichotomously (yes / no) in wave 1 (1996) and wave 2 (2002) To achieve a common metric, variables from wave 3 and 4 were recoded into “yes” (1 = strongly agree, 2 = agree) and “no” (3 = disagree, 4 = strongly disagree) [33].

Previous studies have used similar subjective assessments to identify neighborhood characteristics [36, 37] to investigate the relationship between residential environment indicators and PA.

#### Indicators of individual socioeconomic status

The following facets of SES were assessed: education, household net income, and occupational prestige at follow-up. Education was classified according to the International Standard Classification of Education (ISCED) [38]. In DEAS, a three-level ISCED indicator is available: “low” (ISCED 0–2, pre-primary education up to lower secondary education), “medium” (ISCED 3–4, upper secondary education and post-secondary non-tertiary education) and “high” (ISCED 5–6, first stage of tertiary education up to second stage of tertiary education). Household net income represents income (e.g. wages, pensions) after deduction of taxes and social security contributions. Occupational prestige was defined according to the Standard International Occupation Prestige-Scale (SIOPS) by Ganzeboom and Treiman [39]. SIOPS was operationalized on household level, i.e., the highest-ranking profession was used to indicate household prestige using the five-level scale developed by Hoffmeyer-Zlotnik [40], from one (low) to five (high). For this study these categories were grouped in low (category 1–3: occupations with very little, little or limited autonomy of action) and high (category 4–5: occupations with an independent position or with a high level of autonomy).

#### Confounders and control variables

Gender, time since retirement, and the number of physical diseases from a list of 12 (cardiovascular disease, peripheral vascular disease, back pain, asthma and other respiratory diseases, gut diseases, malignant neoplasms, diabetes, liver or kidney diseases, incontinence, sleep disturbances, visual problems, hearing loss) at follow-up were entered as potential confounders. Previous studies show that changes in PA during the retirement transition differ between men and women [15, 19, 29, 34], and that health restrictions influence participation in PA [41].

### Statistical analyses

Kendall’s rank correlation coefficient was used to examine intercorrelations between individual SES and neighborhood perceptions (Table 2).

Multinomial logistic regression models were used to examine associations between neighborhood factors and interactions of individual SES factors in association with (1) decreases in SA and (2) increases in SA, with the reference group being (0) those who retained their level of activity. For each indicator, odds ratios (OR) of change in SA and corresponding 95% confidence intervals (CI) with adjustment for gender, time since retirement and physical diseases were calculated. Statistical significance was classified with a *p*-value less than 0.05.

The available case analysis was used to deal with missing values of the independent variables in the analysis. All analysis were performed using R version 3.6.1 [42]., with the packages nnorm for multinomial logistic regression and sjplot for graphing significant interactions.

## Results

### Sample demographics

At baseline, the 710 study participants (53% male and 47% female) were on average 60.0 years (SD = 2.4). At the follow-up assessment, participants had been retired for an average period of 2.9 (SD = 3.4) years. On average, they reported 2.1 physical illnesses (SD = 1.6) at follow-up. Almost half the participants had a high level of education (49.3%), and almost everyone else reported a medium (47.2%) level of education (low 3.5%). With regards to SIOPS, 50.2% had a high occupational prestige and 49.8% a low occupational prestige. Average net household income was €3221.76 per month (SD = 1880.5; Table 1). Intercorrelations (Kendall’s τ) between individual SES indicators and neighborhood perceptions were small to moderate in size (Table 2).

**Table 1:** Participant characteristics

Variable	Sample (*n* = 710)
**Age at baseline** (*M* (*SD*))	59.95 (2.38)
**Gender** (*n* (%))	
Male	372 (52.39%)
Female	338 (47.61%)
**Years since retirement at the follow-up assessment** ***(M (SD))***	2.9 (3.39)
**Physical diseases** (*M* (*SD*))	2.07 (1.59)
**Education** (n (%))	
Low	25 (3.52%)
Medium	335 (47.18%)
High	350 (49.30%)
**Household net income in Euro** (*M* (*SD*))	3221.76 (1880.48)
**Occupational prestige** (*n* (%))	
Low	267 (49.81%)
Medium	203 (37.87%)
High	66 (12.31%)
**Positive Memories** (*n* (%))	
Agree	447 (73.39%)
Disagree	162 (26.61%)
**Safety** (*n* (%))	
Agree	515 (84.56%)
Disagree	94 (15.44%)
**Sufficient Facilities Shops** (*n* (%))	
Agree	446 (73.36%)
Disagree	162 (26.64%)
**Sufficient Facilities Health** (*n* (%))	
Agree	519 (85.50%)
Disagree	88 (14.50%)
**Emotional Attachment** (*n* (%))	
Agree	518 (85.20%)
Disagree	90 (14.80%)

Missing data for household net income (*n* = 40), occupational prestige (*n* = 174) physical diseases (*n* = 103), positive memories (*n* = 101), safety (*n* = 101), facilities shops (*n* = 102), facilities health (*n* = 103) and emotional attachment (*n* = 102). Standard deviation (*SD*)

**Table 2:** Intercorrelation matrix (Kendall’s τ) between study variables

	Mean (SD)	1	2	3	4	5	6	7
**1. Occupational prestige**	3.33 (1.09)	1						
**2. Education**	2.46 (0.56)	0.45***	1					
**3. Household income (€)**	3221.76 (1880.48)	0.31***	0.26***	1				
**4. E: Positive Memories**	0.73 (0.44)	−0.02	−0.01	0.06	1			
**5. E: Safety**	0.15 (0.36)	0.01	−0.09*	−0.11***	− 0.04	1		
**6. E: Facilities Shops**	0.73 (0.44)	0.05	0.06	−0.01	− 0.01	0.06	1	
**7. E: Facilities Health**	0.14 (0.35)	−0.11*	−0.02	− 0.08*	0.05	− 0.03	−0.33***	1
**8. E: Emotional attachment**	0.85 (0.36)	−0.09	−0.04	0.01	0.31***	−0.01	−0.03	0.05

Note. E: Environmental perception. * *p* < 0.05; ** *p* < 0.01; *** *p* < 0.001.

### Sports activity

From 710 participants, 383 (53.9%) were classified as being ‘active’ at baseline, and this proportion increased to 443 participants (62.4%) at follow-up. In total, 131 participants (18.5%) increased their SA levels between baseline and follow-up, and 71 (10%) decreased in their SA levels. Correspondingly, SA remained unchanged in 508 (71.6%) individuals.

### Results from multinomial logistic regression

In a first model (Table 3), the main effects of individual SES factors and environmental perceptions was examined. Participants were more likely to decrease their SA compared to retaining their levels of activity if they were female, had lower levels of occupational prestige, felt less attached to their environment, perceived better medical and shop facilities, felt less secure and had more positive memories. Increases compared to retaining previous levels of SA were more likely if participants were female, had higher education, lower occupational prestige, felt more attached and safer in their environment, and perceived lower levels of medical facilities.

**Table 3:** Results from multinomial logistic regression with main effects

Change PA	Predictor	Estimate	Standard Error	***p*** value	95%CI: Lower	95%CI: Upper
Decrease (Reference = No Change in SA)						
	(Intercept)	0.14	0.03	0.00	0.13	0.15
	Number of illnesses	1.07	0.10	0.50	0.88	1.29
	Time since retirement	1.05	0.04	0.20	0.97	1.13
	**Gender**	**0.66**	**0.08**	**0.00**	**0.56**	**0.77**
	**Education**	**1.35**	**0.09**	**0.00**	**1.13**	**1.63**
	Household Income	1.00	0.00	0.50	1.00	1.00
	**Occupational Prestige**	**0.44**	**0.05**	**0.00**	**0.40**	**0.48**
	**E: Attachment**	**0.71**	**0.04**	**0.00**	**0.65**	**0.77**
	**E: Facilities Health**	**1.14**	**0.05**	**0.01**	**1.04**	**1.24**
	**E: Facilities Shops**	**1.29**	**0.07**	**0.00**	**1.12**	**1.49**
	**E. Facilities Safety**	**0.43**	**0.01**	**0.00**	**0.42**	**0.43**
	**E: Positive Memories**	**1.62**	**0.05**	**0.00**	**1.47**	**1.79**
Increase (Reference = No Change in SA)						
	(Intercept)	0.26	0.03	0.00	0.24	0.28
	Number of illnesses	0.95	0.08	0.51	0.81	1.11
	Time since retirement	1.00	0.04	0.91	0.92	1.08
	**Gender**	**0.62**	**0.19**	**0.01**	**0.43**	**0.91**
	**Education**	**1.47**	**0.14**	**0.01**	**1.11**	**1.94**
	Household Income	1.00	0.00	0.08	1.00	1.00
	**Occupational Prestige**	**0.67**	**0.16**	**0.01**	**0.49**	**0.91**
	**E: Attachment**	**2.04**	**0.08**	**0.00**	**1.75**	**2.36**
	**E: Facilities Health**	**0.46**	**0.10**	**0.00**	**0.38**	**0.56**
	E: Facilities Shops	0.95	0.24	0.84	0.60	1.52
	**E. Facilities Safety**	**1.33**	**0.07**	**0.00**	**1.17**	**1.52**
	E: Positive Memories	1.05	0.17	0.76	0.76	1.45

*Note*. E: environmental perception, bold type indicates significant (*p* < .05) parameter estimates

In a second model (Table 4), we examined interactions between education as person-level SES indicator and environmental perceptions. Here, no significant interactions emerged.

**Table 4:** Results from multinomial logistic regressions with interactions of environmental perceptions and level of education

Change PA	Predictor	Estimate	Standard Error	***p*** value	95%CI: Lower	95%CI: Upper
Decrease (Reference = No Change in SA)						
	(Intercept)	0.00	2.54	0.02	0.00	0.40
	Number of Illnesses	1.14	0.09	0.12	0.97	1.35
	Time since retirement	1.06	0.04	0.10	0.99	1.14
	Gender	0.87	0.30	0.63	0.48	1.55
	Education	4.04	0.95	0.14	0.62	26.21
	E: Attachment	1.93	2.03	0.75	0.04	103.63
	E: Facilities Health	8.75	1.93	0.26	0.20	381.07
	E: Facilities Shops	6.65	1.82	0.30	0.19	237.43
	E. Facilities Safety	1.48	1.89	0.84	0.04	59.64
	E: Positive Memories	6.64	1.71	0.27	0.23	189.70
	I: Education*Attachment	0.64	0.77	0.56	0.14	2.88
	I:Education*Health Facilities	0.52	0.78	0.40	0.11	2.42
	I:Education*Shops	0.55	0.72	0.41	0.14	2.24
	I:Education*Safety	0.60	0.82	0.54	0.12	2.99
	I:Education*Memories	0.54	0.65	0.35	0.15	1.96
Increase (Reference = No Change in SA)						
	(Intercept)	0.36	1.77	0.57	0.01	11.57
	Number of Illnesses	1.00	0.07	0.99	0.87	1.15
	Time since retirement	0.98	0.04	0.70	0.91	1.07
	Gender	0.77	0.23	0.24	0.49	1.19
	Education	0.93	0.68	0.91	0.25	3.49
	E: Attachment	2.51	1.70	0.59	0.09	69.95
	E: Facilities Health	0.30	1.74	0.48	0.01	8.98
	E: Facilities Shops	0.25	1.19	0.24	0.02	2.53
	E. Facilities Safety	0.56	1.27	0.65	0.05	6.84
	E: Positive Memories	1.25	1.16	0.85	0.13	12.26
	I: Education*Attachment	0.86	0.65	0.82	0.24	3.05
	I:Education*Health Facilities	1.36	0.68	0.65	0.36	5.17
	I:Education*Shops	1.67	0.47	0.28	0.66	4.19
	I:Education*Safety	1.48	0.50	0.43	0.56	3.93
	I:Education*Memories	0.89	0.45	0.80	0.37	2.16

*Note*. E: environmental perception, I: interaction,, bold type indicates significant (*p* < .05) parameter estimates

**Fig. 2:**
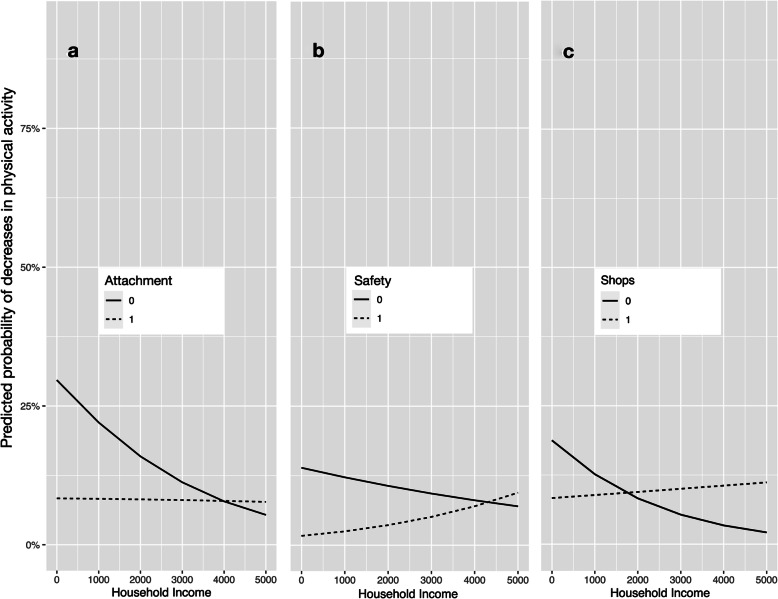
Interactions of household income and environmental perceptions in predicting decreases in sports activity (Reference = No Change)

In a third model (Table 5), we examined interactions between household income and environmental perceptions. In predicting decreases, significant interactions between household income and attachment, perceptions of shops, and perceptions of safety were found. Figure 2 illustrates the shape and direction of these interactions. Figure 2a shows that participants were more likely to decrease their SA if they felt less emotionally attached to their environment and had lower levels of household income – or, put the other way, that household income buffered against the effects of not feeling attached to the neighborhood. Figure 2b suggests that participants who perceived their neighborhood as unsafe were more likely to decrease their activity, but that this effect decreased at higher levels of income. Figure 2c suggests that perceiving little opportunities for shopping in the neighborhood was related to decreases in SA only in participants with lower levels of income.

**Table 5:** Results from multinomial logistic regressions with interactions of environmental perceptions and household income

Change PA	Predictor	Estimate	Standard Error	***p*** value	95%CI: Lower	95%CI: Upper
Decrease (Reference = No Change in SA)						
	(Intercept)	0.82	0.01	0.00	0.80	0.85
	**Number of Illnesses**	**1.19**	**0.07**	**0.01**	**1.04**	**1.36**
	Time since retirement	1.06	0.03	0.08	0.99	1.13
	**Gender**	**0.86**	**0.03**	**0.00**	**0.82**	**0.92**
	**Income**	**1.00**	**0.00**	**0.00**	**1.00**	**1.00**
	**E: Attachment**	**0.17**	**0.01**	**0.00**	**0.16**	**0.17**
	**E: Facilities Health**	**2.96**	**0.00**	**0.00**	**2.95**	**2.97**
	**E: Facilities Shops**	**0.48**	**0.01**	**0.00**	**0.47**	**0.50**
	**E. Facilities Safety**	**0.12**	**0.00**	**0.00**	**0.12**	**0.12**
	**E: Positive Memories**	**1.59**	**0.01**	**0.00**	**1.55**	**1.63**
	I: Income *Attachment	**1.00**	**0.00**	**0.00**	**1.00**	**1.00**
	I: Income *Health Facilities	1.00	0.00	0.18	1.00	1.00
	I: Income *Shops	**1.00**	**0.00**	**0.00**	**1.00**	**1.00**
	I: Income *Safety	**1.00**	**0.00**	**0.00**	**1.00**	**1.00**
	I: Income *Memories	1.00	0.00	0.95	1.00	1.00
Increase (Reference = No Change in SA)						
	(Intercept)	0.43	0.04	0.00	0.40	0.46
	Number of Illnesses	0.99	0.07	0.91	0.87	1.13
	Time since retirement	0.99	0.04	0.87	0.92	1.08
	**Gender**	**0.67**	**0.09**	**0.00**	**0.56**	**0.79**
	Income	1.00	0.00	0.70	1.00	1.00
	**E: Attachment**	**2.83**	**0.04**	**0.00**	**2.63**	**3.04**
	**E: Facilities Health**	**0.43**	**0.00**	**0.00**	**0.43**	**0.43**
	**E: Facilities Shops**	**0.88**	**0.03**	**0.00**	**0.83**	**0.94**
	**E. Facilities Safety**	**2.55**	**0.01**	**0.00**	**2.52**	**2.58**
	**E: Positive Memories**	**0.69**	**0.03**	**0.00**	**0.65**	**0.74**
	I: Income *Attachment	1.00	0.00	0.10	1.00	1.00
	I: Income *Health Facilities	1.00	0.00	0.26	1.00	1.00
	I: Income *Shops	1.00	0.00	0.93	1.00	1.00
	I: Income *Safety	**1.00**	**0.00**	**0.02**	**1.00**	**1.00**
	I: Income *Memories	1.00	0.00	0.12	1.00	1.00

*Note*. E: environmental perception, I: interaction, Bold values indicate statistical significance

**Fig. 3:**
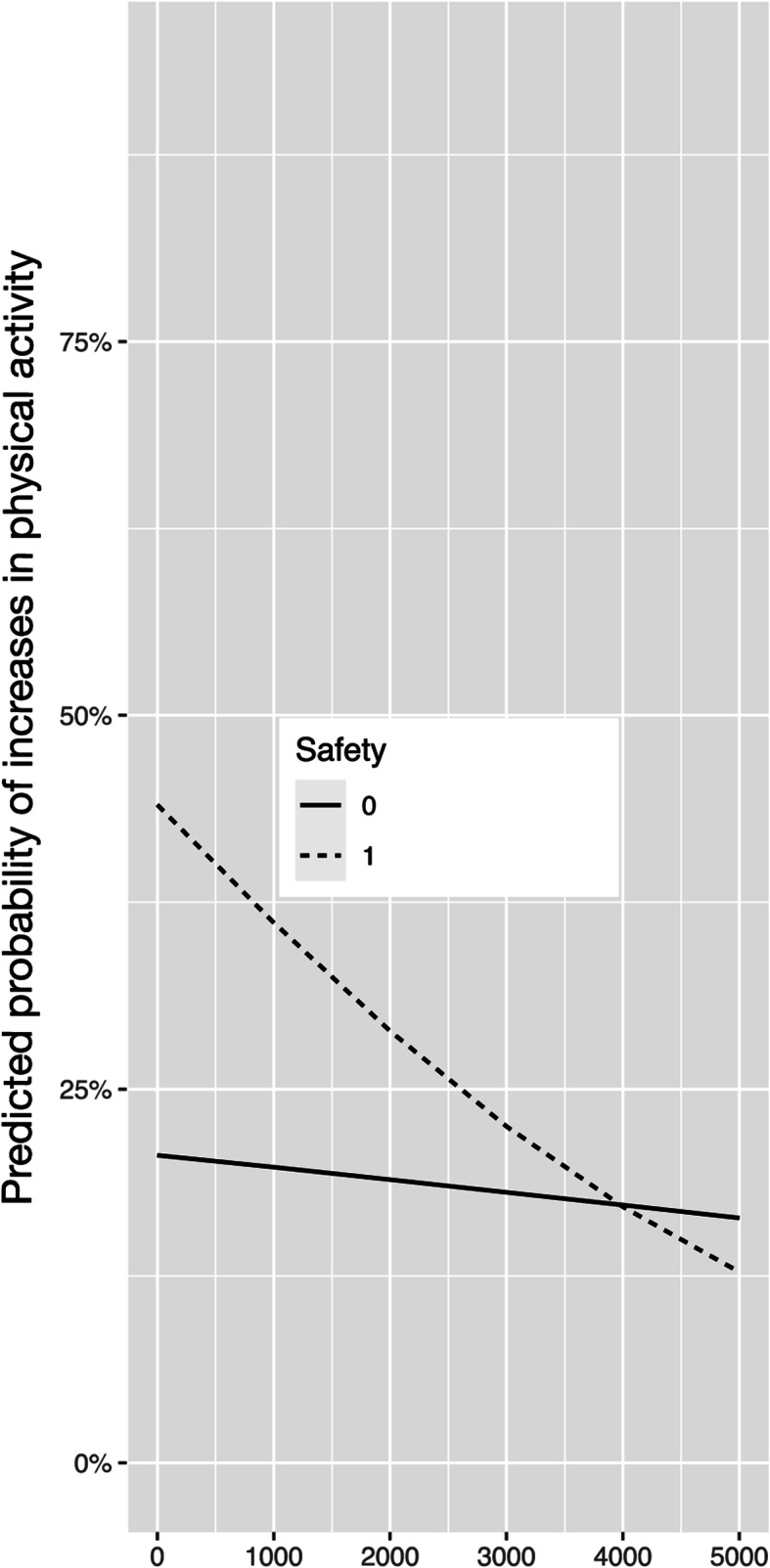
Interaction of household income and safety perceptions in predicting increases in sports activity (Reference = No Change)

For increases in SA, only the interaction between income and safety perceptions was found to be significant. Figure 3 suggests that higher levels of perceptions of safety were related to increases in SAin participants with lower levels of household income.

No significant interactions were found in a fourth model (Table 6) examining interactions between occupational prestige and environmental perceptions.

**Table 6:** Results from multinomial logistic regressions with interactions of environmental perceptions and occupational prestige

Change PA	Predictor	Estimate	Standard Error	***p*** value	95%CI: Lower	95%CI: Upper
Decrease (Reference = No Change in SA)						
	(Intercept)	0.05	1.86	0.10	0.00	1.87
	Number of Illnesses	1.04	0.10	0.68	0.86	1.26
	Time since retirement	1.07	0.05	0.18	0.97	1.17
	Gender	0.68	0.33	0.25	0.36	1.31
	Occupational Prestige	1.49	0.50	0.43	0.56	3.97
	E: Attachment	1.59	1.55	0.77	0.08	33.08
	E: Facilities Health	1.26	1.36	0.86	0.09	18.23
	E: Facilities Shops	2.71	1.18	0.40	0.27	27.55
	E. Facilities Safety	11.16	1.76	0.17	0.35	353.19
	E: Positive Memories	3.81	1.29	0.30	0.31	47.50
	I: Prestige *Attachment	0.76	0.42	0.51	0.33	1.73
	I: Prestige *Health Facilities	0.99	0.45	0.98	0.41	2.36
	I: Prestige *Shops	0.81	0.36	0.55	0.40	1.63
	I: Prestige *Safety	0.27	0.74	0.08	0.06	1.14
	I: Prestige *Memories	0.76	0.37	0.45	0.37	1.56
Increase (Reference = No Change in SA)						
	(Intercept)	0.53	1.59	0.69	0.02	12.10
	Number of Illnesses	0.95	0.08	0.58	0.81	1.12
	Time since retirement	0.99	0.04	0.83	0.91	1.08
	Gender	0.65	0.26	0.10	0.39	1.09
	Occupational Prestige	0.85	0.44	0.71	0.36	2.01
	E: Attachment	1.47	1.42	0.79	0.09	23.83
	E: Facilities Health	0.04	1.62	0.05	0.00	0.97
	E: Facilities Shops	1.17	0.95	0.87	0.18	7.50
	E. Facilities Safety	2.04	1.18	0.55	0.20	20.53
	E: Positive Memories	1.80	0.98	0.55	0.26	12.41
	I: Prestige *Attachment	1.09	0.40	0.84	0.50	2.38
	I: Prestige *Health Facilities	2.19	0.46	0.09	0.88	5.40
	I: Prestige *Shops	0.95	0.27	0.85	0.55	1.63
	I: Prestige *Safety	0.87	0.34	0.70	0.45	1.71
	I: Prestige *Memories	0.86	0.28	0.60	0.50	1.50

*Note*. E: environmental perception, I: interaction, Bold values indicate statistical significance

## Discussion

This study examined potentially modifiable determinants of changes in SA during the transition to retirement based on a social ecological model of activity [19] in a 6-year longitudinal study based on data from the German Ageing Survey (DEAS).

### Changes in sports activity across the retirement transition

The majority of participants retained pre-retirement levels of activity, but a non-trivial proportion of the sample who were previously inactive demonstrated the potential to increase SA during the retirement transition. This points to the potential of this age group to maintain or increase SA, which is particularly relevant before the background of increasing physical inactivity in older adults [43] and sedentary behavior during retirement [41]. These descriptive study results corroborate previous studies. They show that the retirement transition is associated with leisure-time SA [35], with leisure-time PA [17, 30, 44, 45] and with a higher proportion of those who meet the PA recommendations [41]. There are several possible explanations for increases in PA during the retirement transition. On the one hand, greater availability of time and flexibility, and, on the other, changing social networks, support systems and daily routines could facilitate an increase in activity [4, 5]. However, it should be taken into account that this study only examined a maximum period of 6 years. This means the results cannot be generalized to the entire post-retirement period. Other studies [44–46] indicate a temporary increase in leisure-time PA after the retirement transition.

### Individual socioeconomic status and changes in sports activity

SES indicators were relevant for both increases and decreases in SA over retirement: Education and occupational prestige were identified as significant predictors of increases, and in particularly lower occupational prestige was associated with decreases in SA. This suggests that occupational prestige might have a greater association on being active in retirement than just the availability of time. This is in line with previous studies showing change in PA differentiates according to previous occupation [29, 30, 41, 46] and wealth [29]. Qualitative studies indicate that those with a physically demanding job, which is associated with a lower occupational position, describe retirement as a time of well-deserved rest [24]. However, this remains silent to mechanisms underlying changes – thus future studies should address the mechanisms leading to differences in incereases in SA.

### Neighborhood perceptions and changes in sports activity

In this study, we examined both physical and social neighborhood perceptions based on a social-ecological models [19]. In line with previous studies [23–26] and one previous meta-analysis [27], we found neighborhood perceptions to predict both increases and decreases in SA: Higher perceptions of neighborhood safety, higher emotional attachment, but also a lower perception of the availability of medical facilities were related to increases in SA. At the same time, less favorable memories and lower attachment predicted decreases in SA. Interestingly, better perceptions of shopping and medical facilities were also related to decreases in SA. In particular the latter two factors are surprising, as better access to such facilities of everyday life has in previous studies been associated with more PA [47]. One possible explanation is that these factors might not have represented relevant resources for participants. This lack of specific contextual assessments could contribute to inconsistent findings [26]. Future studies should focus in particular on neighborhood-related SA to avoid an environment–behavior mismatch [26].

### Interactions of individual socioeconomic and neighborhood factors

According to a social ecological model [19, 28–30], this study examined interactions between individual SES and perceptions of the neighborhood environment in predicting change in SA.

We found no interactions between education or occupational prestige and environmental perceptions. This result appears promising, implying that neighborhood environmental properties are equally important for SA regardless of socioeconomic position. A previous study [27] also found no significant interaction between education and residential environment-related indicators at the beginning of retirement.

We did find interactions between household income and environmental perceptions, however. Lower attachment predicted decreases in participants with lower income – suggesting that these participants might be at additional risk for decreases in activity over the retirement transition if they live in neighborhoods that provide few anchor points for attachment. Higher perceptions of safety buffered against decreases in those with lower income, suggesting that this might be an important resource in particular in lower-SES neighborhoods – or that those with higher incomes can afford to travel to engage in activity, e.g., in sports clubs [48]. Income also moderated the relationships between perceptions of shop facilities and decreases in SA such that lower perceptions of shopping facilities were associated with a lower likelihood of decreases in those with higher income, which could be due to a lower density of shops and medical facilities in more affluent suburban neighborhoods which in turn would provide more opportunities for SA.

Similar to the findings for decreases in SA, we found an interaction between income and safety perceptions and increases in activity such that in particular in participants with lower income, higher safety perceptions were associated with a higher predicted probability of increases in SA. Together, these findings for safety suggest that perceptions of safety are a key resource both for preventing decreases in activity and in promoting increases in SA in older adults, particularly in those with lower incomes. This finding also replicates previous studies on the role of neighborhood safety perceptions in PA in older adults [49–51], but extends these findings to the notion that safety perceptions might be particularly important in older adults with lower incomes.

### Strengths and limitations

The transition to retirement represents a key transition in later adulthood and has not yet been sufficiently investigated with regards to changes in SA [27]. One strength of this study is the use of DEAS data with low sample selectivity and a distribution of socio-demographic characteristics that is in line with the German population [52]. However, panel selectivity limits generalization - panel participants are younger, healthier, more educated, have a higher income and larger informal networks compared to participants who dropped out [52]. A further strength of this study is the longitudinal design which goes beyond previous cross-sectional studies on neighborhood environment indicators and leisure-time PA [27].

There are some limitations to the current study that limit the interpretation of the findings. First, all analyses are based on self-reported SA [52], thus overestimation of SA behavior due to recall and information bias [53] or social expectation bias [54] cannot be ruled out. The assessment of SA with a single item further potentially limits reliability and validity of the measurement due to measurement error. Assuming that these systematic errors had the same impact at baseline and follow-up assessment however, the analysis should remain unaffected. In addition, this study did not examine duration and intensity, but only broad categorical information about the frequency of SA, which differentiates between active and inactive people. No statement can be made about the exact extent of changes in SA. Considering that the WHO [55] recommends at least 150 min of moderate PA or at least 75 min of vigorous or SA per week, it would be of particular public health interest to examine associations for different intensities of PA at the retirement transition.

Furthermore, there is substantial heterogeneity in how individuals realize the retirement transition [56]. Because previous studies showed differences in the change of leisure-time PA after the retirement transitions (e.g. due to illness; 18, 46), the results of this study are not transferable to the entire retiree population.

Another limitation constitutes the neighborhood variables, which are based on self-reports using single items with the associated limitations in reliability and validity due to the inability to control for measurement error. Even if a wide range of environmental aspects could be examined, the possibility of a discrepancy between perception and reality must be taken into account including over or under reporting biases [26]. Perceived environmental characteristics differ from objective survey methods [57], which suggests that the use of objective methods, such as geographic information system (GIS) might provide different findings than subjective perceptions.

## Implications and conclusions

Despite these limitations, the findings from this study suggest that in addition to known SESfactors, in particular perceptions of the safety of the neighborhood predict positive and negative changes in SA during the retirement transition. The results produced supplement the existing literature and should be considered when planning interventions to prevent physical inactivity in older adults. The results of this study are in line with social ecological models that describe several levels of influence on health-related behavior [19, 21]. According to this model, it would also be of further interest to investigate associations at the levels of politics and culture [19, 21].

## Data Availability

This study is based on data from the public release of the German Ageing Survey (DEAS), provided by the Research Data Centre of the German Centre of Gerontology (DZA) and funded by the Federal Ministry for Family Affairs, Senior Citizens, Women and Youth (BMFSFJ). The analysis refers to the following datasets: SUF DEAS 2017, version 1.0, DOI: 10.5156/DEAS.2017.M001. Web link: https://www.dza.de/en/research/fdz/german-ageing-survey/data/data-of-survey-year-2017, SUF DEAS 2014, version 2.0, DOI: 10.5156/DEAS.2014.M.001. Web link:https://www.dza.de/en/research/fdz/german-ageing-survey/data/data-of-survey-year-2014, SUF DEAS 2011, version 2.1, DOI: 10.5156/DEAS.2011.M.004. Web link:https://www.dza.de/en/research/fdz/german-ageing-survey/data/data-of-survey-year-2011, SUF DEAS 2008, version 3.1, DOI 10.5156/DEAS.2008.M.005. Web link: https://www.dza.de/en/research/fdz/german-ageing-survey/data/data-of-survey-year-2008, SUF DEAS 2002, version 3.1, DOI 10.5156/DEAS.2002.M.005. Web link: https://www.dza.de/en/research/fdz/german-ageing-survey/data/data-of-survey-year-2002 and SUF DEAS 1996, version 3.0, DOI 10.5156/DEAS.1996.M.004. Web link: https://www.dza.de/en/research/fdz/german-ageing-survey/data/data-of-survey-year-1996. DEAS data is available for research purposes after signing a user contract. R code and output for the current manuscript is available as supplementary material online.
